# The Biomechanical Function of Periodontal Ligament Fibres in Orthodontic Tooth Movement

**DOI:** 10.1371/journal.pone.0102387

**Published:** 2014-07-18

**Authors:** Steven W. McCormack, Ulrich Witzel, Peter J. Watson, Michael J. Fagan, Flora Gröning

**Affiliations:** 1 Medical and Biological Engineering Research Group, School of Engineering, University of Hull, Hull, United Kingdom; 2 Fakultät für Maschinenbau, Ruhr-Universität Bochum, Bochum, Germany; 3 Musculoskeletal Research Programme, School of Medicine and Dentistry, University of Aberdeen, Aberdeen, United Kingdom; Ohio State University, United States of America

## Abstract

Orthodontic tooth movement occurs as a result of resorption and formation of the alveolar bone due to an applied load, but the stimulus responsible for triggering orthodontic tooth movement remains the subject of debate. It has been suggested that the periodontal ligament (PDL) plays a key role. However, the mechanical function of the PDL in orthodontic tooth movement is not well understood as most mechanical models of the PDL to date have ignored the fibrous structure of the PDL. In this study we use finite element (FE) analysis to investigate the strains in the alveolar bone due to occlusal and orthodontic loads when PDL is modelled as a fibrous structure as compared to modelling PDL as a layer of solid material. The results show that the tension-only nature of the fibres essentially suspends the tooth in the tooth socket and their inclusion in FE models makes a significant difference to both the magnitude and distribution of strains produced in the surrounding bone. The results indicate that the PDL fibres have a very important role in load transfer between the teeth and alveolar bone and should be considered in FE studies investigating the biomechanics of orthodontic tooth movement.

## Introduction

Bone mass and structure is thought to be adapted to its mechanical environment [1]. One important clinical consequence of mechanical adaptation of bone is orthodontic tooth movement, which occurs due to site-specific resorption and formation of alveolar bone [2]. The crown loading conditions required to move teeth during orthodontic treatment are reasonably well understood [3], typically with low continuous forces of around one newton applied for weeks at a time [4]. Load transfer from the teeth to the surrounding bone is influenced by the periodontal ligament (PDL), which is the fibrous connective tissue that fills the space between the tooth root and alveolar bone. Whilst bone remodelling has been widely investigated in long bones, the presence of the PDL makes direct application of these theories to alveolar bone remodelling more difficult.

**Figure 1:**
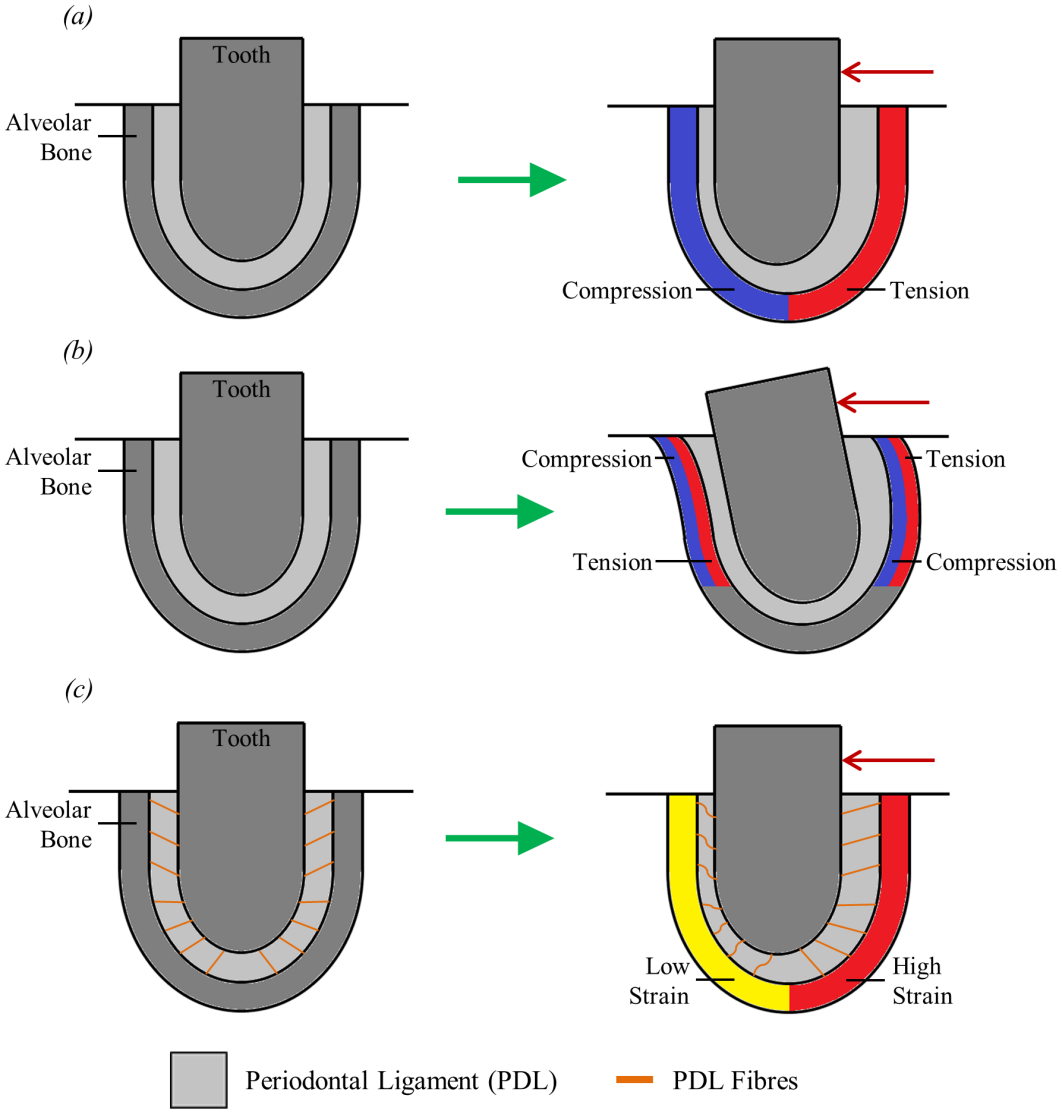
Simplified two dimensional representation of a tooth-PDL-bone complex illustrating three hypotheses for strain-based bone remodelling during orthodontic tooth movement. (a) “pressure-tension hypothesis” showing tooth displacement leading to compression and tension in the surrounding bone; (b) “alveolar bending hypothesis” showing tooth movement causing bending of the alveolar bone; (c) “stretched fibre hypothesis” showing stretching and compression of PDL fibres leading to low and high strain areas in the surrounding bone. The red arrows indicate the direction of the applied orthodontic force.

A number of different hypotheses have been suggested regarding the biomechanical nature of orthodontic tooth movement. One of the oldest hypotheses is known as the “pressure-tension hypothesis”. This suggests that tooth movement in the direction of applied load compresses the PDL on the side to which the tooth is moved and stretches it on the opposite side. This leads to symmetric zones of compression and tension occurring in the periodontium (Fig. 1a), with the compression leading to bone resorption and tension causing bone formation [2], [5]. However, this hypothesis is not in agreement with how bone adaptation is generally understood. Here, an increase in loading and thus bone strain is related to bone formation whereas a decrease in loading is thought to cause bone resorption (e.g. Melsen [6]). To test this pressure-tension hypothesis Cattaneo *et al*. [5] developed a finite element (FE) model to examine the stress distribution in the periodontium after the application of an orthodontic load. They concluded that orthodontic tooth movement could not be explained simply by compression and tension in the direction of the applied load.

A second hypothesis regarding orthodontic tooth movement is the “alveolar bending hypothesis” first reported by Baumrind [7]. This suggests that as well as deforming the PDL, tooth movement also causes deformation of the alveolar bone. In this hypothesis (Fig. 1b), the walls of the tooth socket behave like cantilever beams, that is, they are essentially fixed at one end (towards the apex) and free at the other (towards the tooth crown). When an orthodontic load is applied, this displaces the free end and, since the other end is fixed, a slight bending of the tooth socket walls occurs. The bone on the side to which the tooth is pushed is bent away from the tooth and the bone on the other side is pulled towards the tooth.

The alveolar bending hypothesis explains bone remodelling building on an idea initially proposed by Frost [8], and further developed by Currey [9]. According to Currey [9] strain gradients are responsible for determining the nature of bone adaptation where bone is added to a surface if, under the application of a load, the strains in the bone become more tensile with depth from the surface. Conversely, bone is removed if strains become less tensile with depth. Applying this idea to the alveolar bending hypothesis (Fig. 1b), bone would be added to the compressive surfaces of the alveolar bone and removed from the tensile surfaces causing the position of the tooth to move in the direction of the applied load.

Recently, a third hypothesis has been suggested by Melsen [6] which is intended to match orthodontic tooth movement with orthopaedic bone remodelling in accordance with Frost's mechanostat theory [10], in which low strain leads to bone resorption and high strain leads to bone formation. The hypothesis suggested by Melsen [6] is the “stretched fibre hypothesis”. Typically teeth move in the direction of the applied force. The pressure-tension hypothesis assumes that displacement of the tooth in its socket compresses the alveolar bone in that direction and this bone is then resorbed and new bone is formed on the opposite side (Fig. 1a). However, Melsen [6] suggests that this might not be the case due to the elastic fibres in the PDL. These PDL fibres will be compressed on the side to which the tooth is pushed, and stretched on the opposite side (Fig. 1c). Thus the PDL fibres will only exert force on the surrounding bone where they are in tension, not where they are in compression. Therefore, the PDL will provide little resistance to tooth movement in the direction of the applied force and so will transfer negligible load to the alveolar bone on that side. Conversely, the fibres will be stretched on the opposite side and thus the applied load will be essentially transferred there. The mechanostat theory can then be used to explain orthodontic tooth movement: under-loading of the alveolar wall causes bone resorption, while the load exerted by the stretched PDL fibres on the opposite side causes bone formation. If this is correct, then it would be important to include the fibres of the PDL in FE models, especially when investigating orthodontic tooth movement. However, due to the nature of these fibres it is difficult and time-consuming to model them. Therefore, careful consideration should be given as to whether it is necessary to do so.

Whilst the deformation of the alveolar bone may have an important role in the mechanism responsible for orthodontic tooth movement, it has also been widely suggested that orthodontic tooth movement is actually mediated by the PDL rather than the alveolar bone (e.g. Toms *et al*. [11]). The primary reason for this suggestion is that whilst large strains can occur in the PDL, strains in the alveolar bone due to orthodontic loading are thought to be too low to typically cause remodelling to occur (e.g. Chen et al., [12]). This has been reinforced by several studies involving FE models which have predicted strains in the alveolar bone far below what is generally thought to cause bone remodelling (e.g. [3], [13]–[15]). However, none of these studies have included the fibrous structure of the PDL in their FE models, which may be important when considering the strain in the PDL and alveolar bone. It has also been observed that when loads are applied to teeth without a PDL only a limited amount of bone remodelling occurs, which further suggests that orthodontic tooth movement is controlled by the PDL [16].

The PDL contains elastic fibres (mainly collagen) surrounded by a matrix of other components such as blood and lymph vessels, and interstitial fluid [17]. It is approximately 0.25 mm±50% wide with the fibres making up typically fifty to seventy-five per cent of the tissue volume [18], [19]. The collagen fibres are grouped together in principal fibre bundles and form a meshwork similar to a stretched fishing net extending between the cementum and alveolar bone [16], [20], [21]. The complex arrangement of fibres ensures that regardless of the direction of force applied, some fibre bundles are always placed in tension [21]. The fibres are also thought to transmit vertical forces from the teeth as lateral forces to the tooth socket and in doing so, help to prevent high stresses occurring at the apex of the tooth root [22]. The PDL has been shown to have nonlinear, viscoelastic material properties which vary at different locations and in different directions along the tooth root [2], [17], [21]. However, whilst attempts have been made to characterise the material properties of the whole system, little is known about the specific material properties, or geometry, of the individual PDL fibres [23].

When simulating masticatory and orthodontic loads with an FE model of the mandible or cranium, the way in which the PDL is modelled can have a significant influence on the results produced. Whether or not to include the PDL in such FE models, as well as what material properties to assign, is the subject of much debate throughout the literature (e.g. [24]–[26]). Most FE models of the masticatory apparatus idealise the PDL as a layer of solid, homogeneous and isotropic material. Although some authors have attempted to represent its material properties more accurately, only a few have attempted to include its fibre-reinforced structure [23], [27]–[31]. Some of these [23], [28], [31] have investigated orthodontic tooth movement, but did not focus on the mechanical stimuli for alveolar bone remodelling. Other authors have examined orthodontic bone remodelling (e.g. Cattaneo et al. [2]) but did not include the fibrous structure of the PDL.

Here we present the first FE analysis of orthodontic bone remodelling which takes into consideration the fibrous structure of the PDL. We developed a simplified three-dimensional single tooth FE model to analyse the effect of including the PDL fibres. Using the same basic model, two different representations of the PDL were tested: solid PDL and fibrous PDL. Three different loads were then applied to each of the two models and the strains produced in the surrounding bone compared. The results from this single tooth model give an indication as to whether or not the increased time and effort required to include PDL fibres is justified, and whether the bone strains predicted by a fibrous PDL model are consistent with current hypotheses about bone remodelling in orthodontic tooth movement.

## Materials and Methods

### 2.1. FE model creation

**Figure 2:**
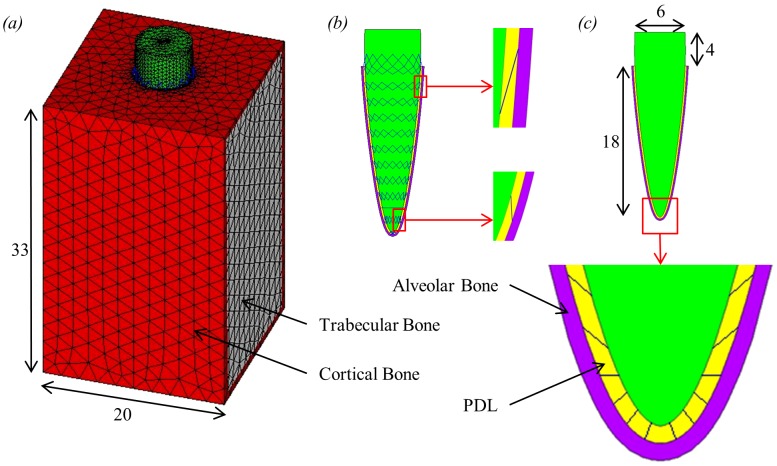
Single tooth FE model including dimensions in millimetres. (a) the whole 3D single tooth model; (b) section through tooth, PDL and alveolar bone showing the location of the link elements which span the PDL layer connecting the tooth and alveolar bone; (c) section through the centre of the model showing the tooth, PDL and alveolar bone including an expanded view of the apex region.

An idealised single tooth FE model was created using ANSYS software (version 13.0, ANSYS Inc., Canonsburg, PA, USA). The size and shape of the model, shown in figure 2, were chosen to be comparable to a human tooth and are in keeping with similar models (e.g. Katona & Qian [29]). The tooth root is surrounded by a uniform layer to represent the PDL, which is in turn surrounded by another uniform layer to represent the alveolar bone, both 0.2 mm thick [29], [32]. The tooth, PDL and alveolar bone components were then surrounded by a block to represent the mandibular bone.

The tooth and alveolar bone volumes were meshed with 10-noded higher order tetrahedral structural solid elements (SOLID187). The mandibular bone block was modelled as trabecular bone surrounded by a 2.5 mm thick layer of cortical bone, as shown in figure 2. The trabecular bone was meshed with the same elements as the tooth and alveolar bone (SOLID187) whereas the surrounding cortical bone was meshed using 6-noded triangular structural shell elements (SHELL281).

Two different models were created, varying only in how the PDL was modelled: a solid PDL model and a fibrous PDL model. In the solid PDL model, the PDL volume was again meshed with the same 10-noded elements (SOLID187). Within the fibrous PDL model, to represent the fibre-reinforced matrix structure, the volume of the PDL was first meshed the same as for the solid PDL model. The PDL fibres were then added to the model using tension-only 3D spar elements (LINK10). These link elements connected nodes on the junction between the alveolar bone and PDL with nodes on the junction between the tooth root and PDL. In reality, these fibres show a complex arrangement throughout the PDL, however, in this model the fibres were given the simplified structure shown in figure 2. In total, both models contained 101,326 higher order tetrahedral elements and 1,528 shell elements. The fibrous PDL model also had an additional 448 link elements.

The material properties assigned to the fibrous PDL model were obtained from previous studies (Table 1). The tooth was not separated into different components and was modelled entirely as dentin. This simplification has been used in previous studies (e.g. [35], [36]) and is justifiable since it is strain in the bone (rather than the tooth) which is of interest here. The tooth, PDL and bone were all assumed to be homogeneous, isotropic and linear elastic materials. Although bone is known to have anisotropic material properties it is commonly simplified to isotropic in FE models and validation studies have determined that meaningful results can still be obtained from isotropic models (e.g. [37]–[39]). Similarly, the PDL is known to have nonlinear material properties yet most previous models which include the PDL, model it as a linear elastic material [40].

**Table 1:** Mechanical properties assigned to each material in the fibrous PDL model.

Component	Young's Modulus (MPa)	Poisson's Ratio
Trabecular bone^a^	56	0.30
Cortical bone^b^	17 000	0.30
Tooth^b^	17 000	0.30
PDL matrix^c^	1	0.45
PDL fibres^d^	1 000	0.35

a Misch *et al*. [33].

b Gröning *et al*. [24].

c Jones *et al*. [15], Qian *et al*. [23].

d Gautieri *et al*. [34], Katona and Qian [29], Meyer *et al*. [31], Rees and Jacobsen [18].

For the PDL fibres, as well as defining the mechanical properties, it was necessary to specify the cross-sectional area and initial strain of the link elements. Approximately 50 to 70% of the PDL tissue volume is made up of the PDL fibres [19]. Therefore, the cross-sectional area of the link elements was calculated so that the combined volume of the link elements would be between 50 and 70% of the PDL volume in the model. From this the cross-sectional area was chosen to be 0.06 mm^2^. This value makes the link elements in the model one to two orders of magnitude thicker than the collagen fibres found in a real PDL [21], [31]. This is due to the fact that the number of modelled PDL fibres is well below the number of fibres in the real PDL. No reliable data was available to suggest a suitable value for any initial strain of the link elements and so it was defined as zero.

The solid PDL model was constructed with the same method as the fibrous PDL model, except it did not include the link elements representing the PDL fibres. In order to make a fair comparison between the two models, it was necessary to adjust Young's modulus value given to the PDL component of the solid PDL model, while all other material properties remained the same. This value was adjusted so that the overall effective elastic modulus of the PDL layer in the two models was the same. From this it follows that any differences observed in the stress and strain values between the solid PDL and fibrous PDL models could be attributed to the structural difference, i.e. the presence or absence of PDL fibres, between the two models (rather than to the difference in the elastic modulus of the PDL) [22].

As a criterion for adjusting Young's modulus we used tooth displacement, i.e. the two models were assumed to have the same overall effective elastic modulus when they showed the same tooth displacement under identical loading conditions. Tooth displacement was defined as the change in distance between the most apical node on the tooth root and the corresponding node on the alveolar bone [29]. Thus, under a load of 500 N, the tooth displacement for the fibrous tooth model was 0.0703 mm. This value would seem reasonable when compared to the vertical intrusion of 0.12 mm cited by Borák *et al*. [32] from an experiment by Kato [41]. To get the same displacement for the solid PDL model, it was found necessary to use a Young's modulus value of 12.2 MPa for the PDL layer (determined through the ANSYS optimisation facility).

**Figure 3:**
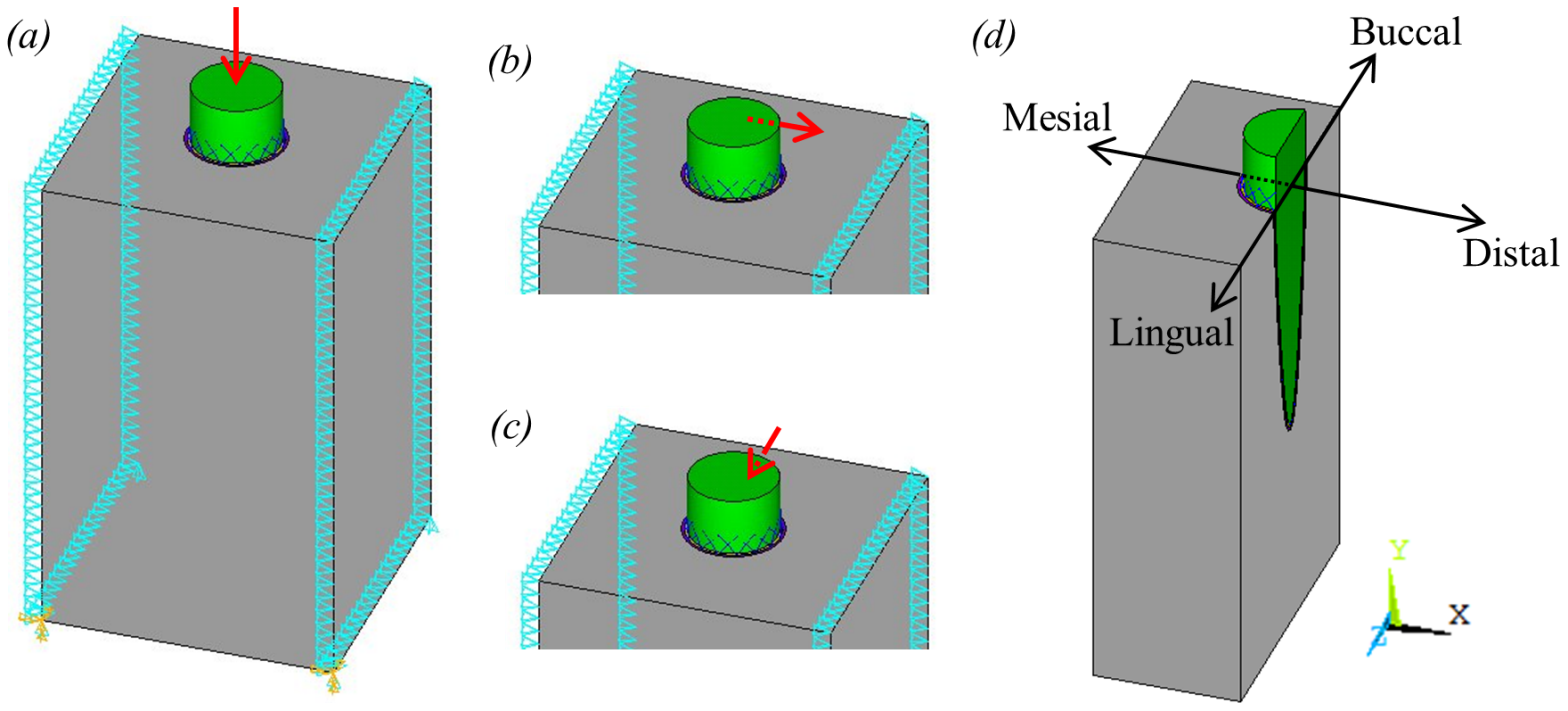
Tooth model showing loading and boundary conditions applied, where triangles represent constraints and red arrows represent applied forces. (a) vertical occlusal load; (b) buccolingual orthodontic load; (c) mesiodistal orthodontic load; (d) section through centre of tooth model showing a typical plane from which strain results were taken.

In single tooth FE models, one common way to apply the boundary conditions is simply to fix all nodes on the two opposing sides of the model to which the rest of the mandible would be connected (e.g. Qian *et al*. [23]). However, a number of studies have reported that over-constraining a model can lead to inaccurate results (e.g. Marinescu *et al*. [42]). Therefore, this method was not used here. Instead, the boundary conditions illustrated in figure 3 were applied. Briefly, all of the nodes around the edge of these two sides were constrained in the x-direction (mesiodistal, figure 3) to represent the increased stiffness in this location due to the presence of cortical bone. The nodes on the rear corners on the base of the model were also constrained in the y-direction (coronoapical) and the nodes on the front corners of the base were constrained in all degrees of freedom. This prevented rigid body translation of the model, while still allowing some deformation in the z-direction (buccolingual).

Three different types of load were applied separately to the model: a 500 N occlusal load and two different 1 N orthodontic loads. The occlusal load was applied as a single vector, point load, acting on the node at the centre of the tooth crown, parallel to the long axis of the tooth. Although occlusal loads may be more complex than this, we are not interested in different loading patterns or the strain distribution through the tooth itself, hence this simplification was considered acceptable in this current analysis. A load of 500 N was chosen to represent a maximal human bite force [43], [44]. To simulate an orthodontic load, a point force was applied to the node half way between the top of the bone and the top of the tooth on the far side of the tooth, as shown in figure 3. The load applied was chosen to be 1 N to represent a typical load used during orthodontic treatment [45]. Two different orthodontic loads were simulated by varying the direction of the force applied to the node: a mesiodistal load and a buccolingual load.

### 2.2. Model testing

In order to assess the reliability of the results produced from these models a number of tests were carried out. First, although the PDL is usually included in single tooth models, some models do not include it, especially models of whole mandibles or crania (e.g. [42], [46]). So, for completeness, a model without a PDL was developed. Following this, a simplified U-shaped model of a whole mandible was created to test the effect of the boundary conditions on the results around the alveolar bone. The original models were also remeshed with much smaller elements to determine whether or not the results had converged. Since this is a very simplified model, direct validation against experimental strain results is not possible. Therefore, to validate the model, tooth displacement results were compared to previously published results. Finally, a sensitivity analysis was carried out to assess the effect of altering the material properties assigned to the fibrous PDL. More details of this model testing are provided in the supplementary material (see File S1).

### 2.3. Solid PDL versus fibrous PDL

The aim of this study was to investigate the effect of including the fibrous structure of the PDL in FE models when applying either occlusal or orthodontic loads. After all model testing was completed, the results obtained from the solid PDL model were compared to the results obtained from the fibrous PDL model. To provide an initial comparison of the results contour plots were produced so that the magnitude and distribution of strains in the two models could be compared by eye. Following this, to provide a more precise comparison of the results, nodal strain graphs were plotted.

Nodal strain results were taken from three regions within the model: the inside surface of the alveolar bone (i.e. adjacent to the PDL), the outside surface of the alveolar bone (i.e. adjacent to the trabecular bone) and a line through the trabecular bone itself. For the occlusal load and buccolingual orthodontic load the results were taken along a line of nodes though the centre of the tooth model in the buccolingual direction (see figure 3). Similarly, for the mesiodistal orthodontic load the results were taken along a line of nodes through the centre of the tooth model in the mesiodistal direction. The FE mesh was identical for both the solid and fibrous PDL models and so the same nodes were used to plot the results for both models. Results were not plotted at nodes which were connected to link elements. For the line through the trabecular bone, suitable nodes were selected (approximately 1 mm from the alveolar bone) by eye since there was no smooth line of nodes through this region. However, since these nodes were the same in all models, the strain distributions could still be compared with confidence.

Maximum and minimum principal strains were extracted along each of these regions for both solid and fibrous PDL models and these results were plotted against each other for visual comparison. For the solid PDL model, the nodal results were plotted and a smooth line was drawn connecting the points. However, for the fibrous PDL model, strain results obtained at the inside and outside surfaces of the alveolar bone were not smooth but rather contained fluctuating values due to strain concentrations developing at the location of the link elements. In a real PDL these high peak strains would not develop due to the much greater number of PDL fibres than represented in this model which would cause a smoother distribution of strain. Since these peak strains are not biologically realistic, rather than simply connecting the points, a third order trend line was plotted through the data to smooth out the results. This was performed for the results from the inside and outside surface of the alveolar bone for the fibrous PDL model in all three load cases. This smoothing was not necessary for the trabecular bone nodal results from the fibrous PDL model, as these nodes were sufficiently far away from the location of the link elements to not be effected by the local stress concentrations.

## Results

### 3.1. Model testing results

As well as testing the results from a no PDL model, four key issues were investigated with respect to model testing: boundary conditions, mesh convergence, model validation and sensitivity to changes in the PDL. The no PDL model showed that the PDL is important in order to reduce the local bone strain in the region surrounding the tooth root (see Fig. S1 in File S1). The results from the U-shaped model showed that the boundary conditions chosen for the single tooth model were acceptable since they produced very similar results (see Table S1, Fig. S4, and Fig. S5 in File S1). The mesh was shown to have converged and the tooth displacement results were seen to be of similar magnitude to previously reported results (see File S1 for details). The sensitivity analysis revealed that adjusting the material property values assigned to the PDL can have a substantial influence on the results obtained (see Table S2 in File S1). Further details of the model testing can be seen in the supplementary material (see File S1).

### 3.2. Solid PDL versus fibrous PDL results

**Figure 4:**
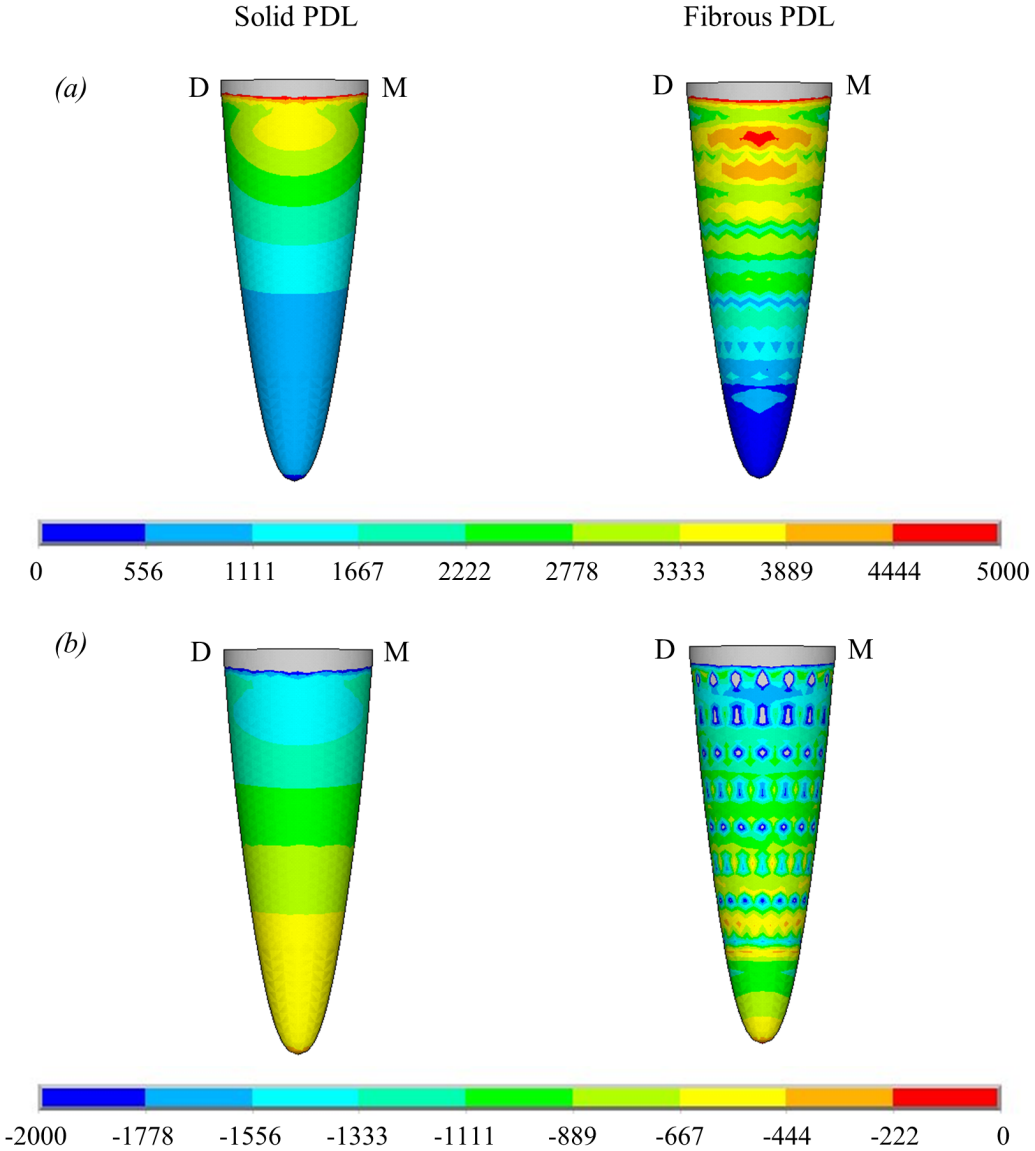
Contour plots showing nodal solutions for the principal microstrains on the outside surface of the alveolar bone for the solid PDL and fibrous PDL models due to the 500 N occlusal load. (a) maximum principal microstrain; (b) minimum principal microstrain. Directions are represented by M for mesial and D for distal.

**Figure 5:**
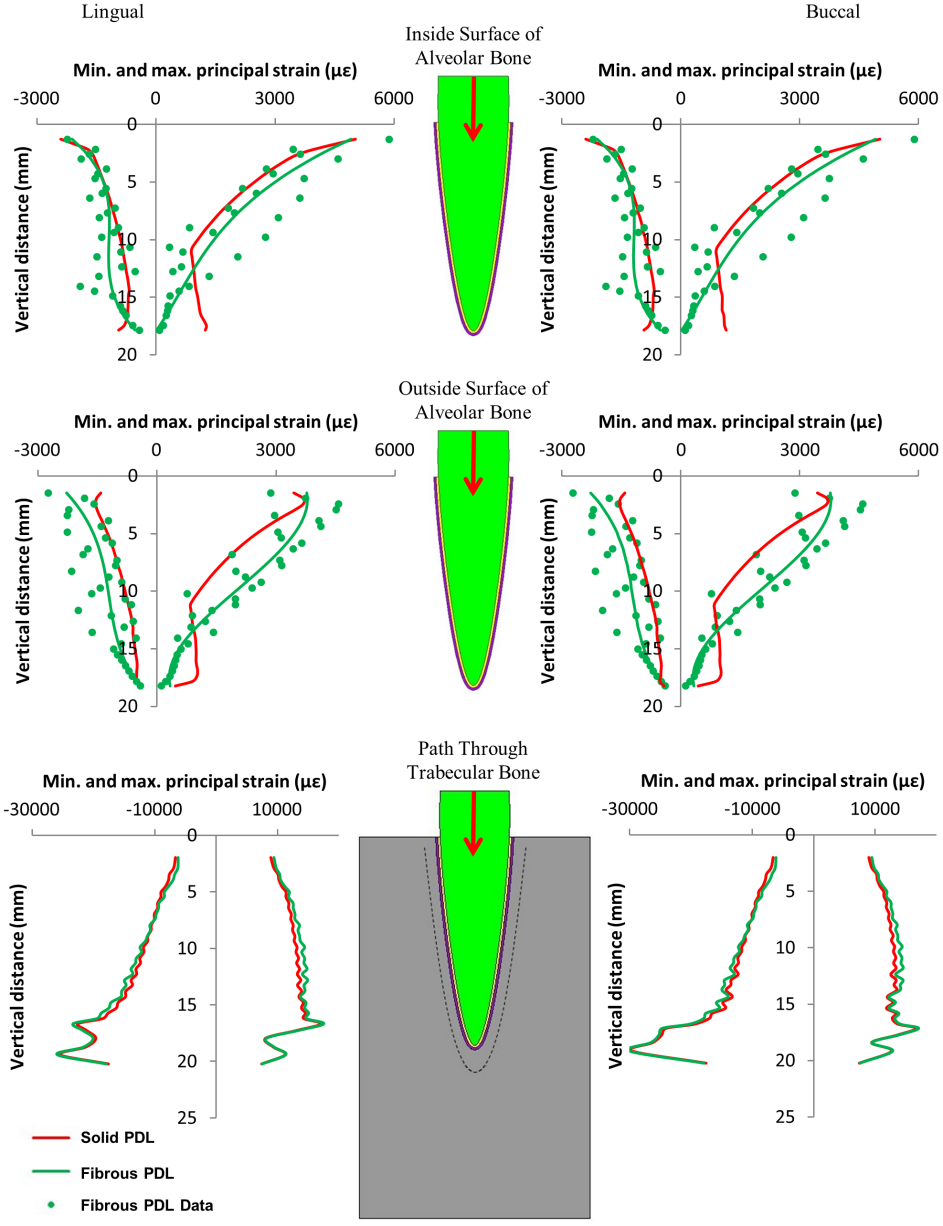
Maximum and minimum principal strains on the buccal and lingual sides of the tooth model from the 500 N occlusal load, along three regions within the model. The graphs relate strain to the vertical distance away from the top of the tooth socket.

**Figure 6:**
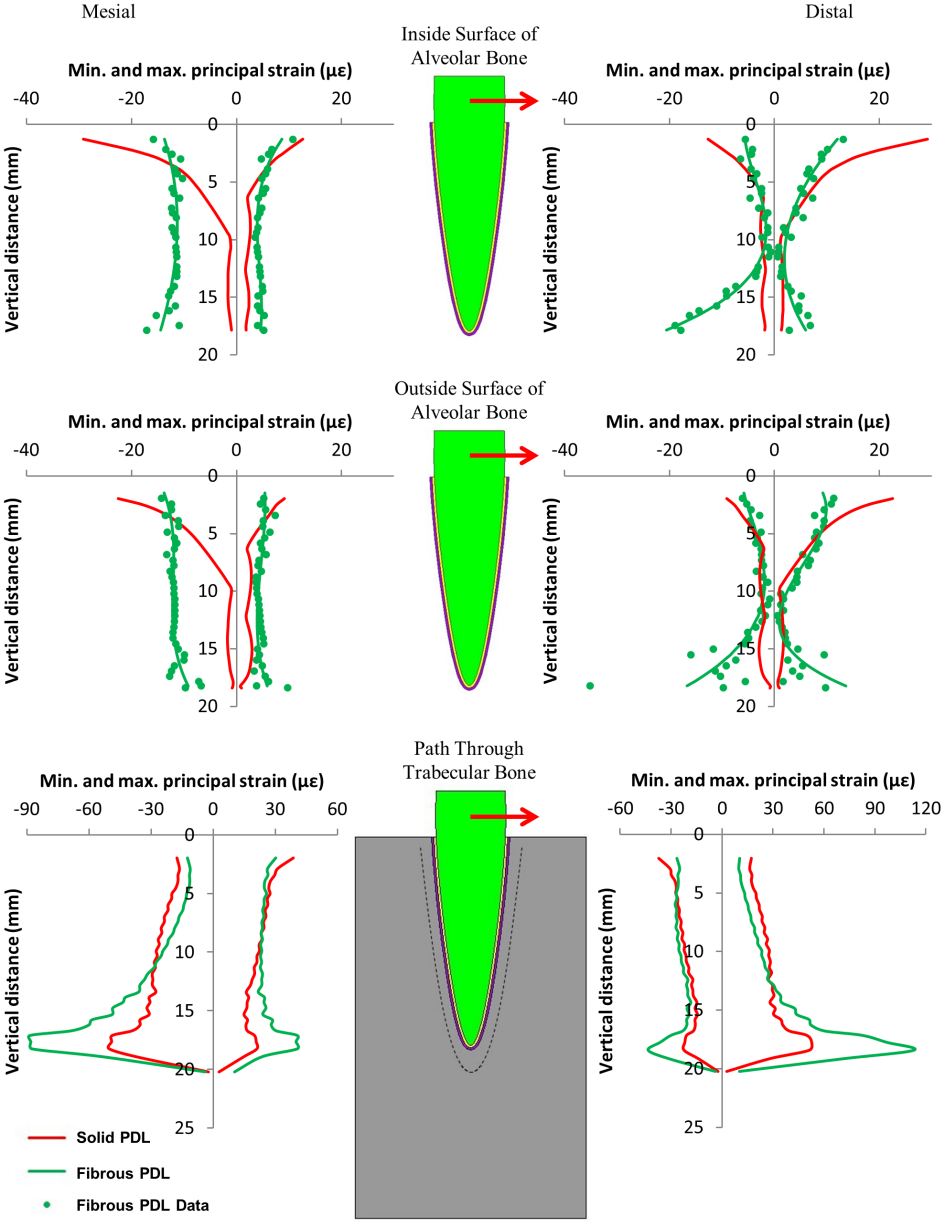
Maximum and minimum principal strains on the mesial and distal sides of the tooth model from the 1 N orthodontic load in the mesiodistal direction, along three regions within the model. The graphs relate strain to the vertical distance away from the top of the tooth socket.

**Figure 7:**
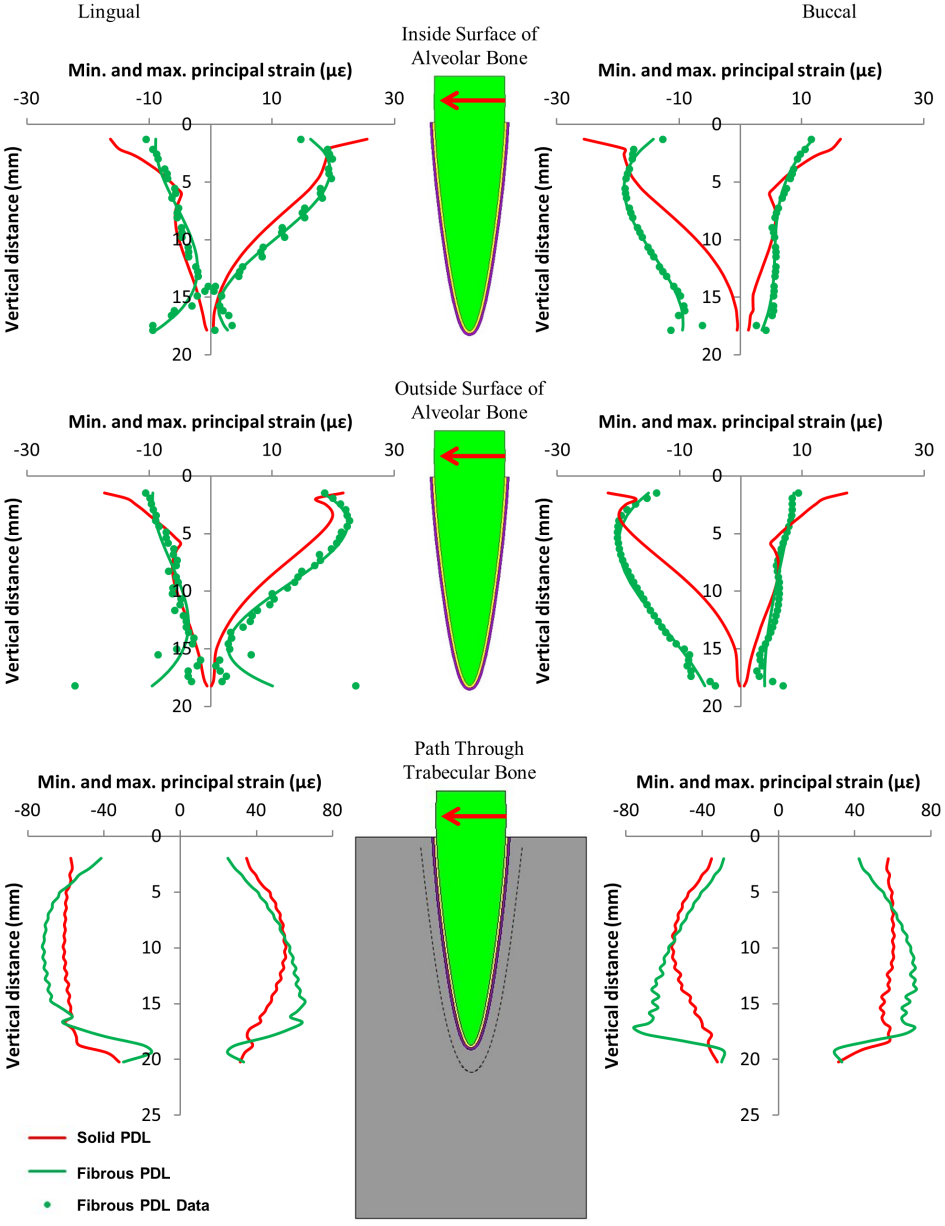
Maximum and minimum principal strains on the buccal and lingual sides of the tooth model from the 1 N orthodontic load in the buccolingual direction, along three regions within the model. The graphs relate strain to the vertical distance away from the top of the tooth socket.

Contour plots of maximum and minimum principal strain in the alveolar bone due to the 500 N occlusal load are shown in figure 4. These contour plots show that omitting the fibrous structure from the PDL does have an effect on the distribution of strain in the alveolar bone. Figure 4 shows strain on the outside surface of the alveolar bone. Although the link elements are only attached at the inside surface of the alveolar bone, high strain concentrations around the link elements are clearly visible. To remove the effect of these strain concentrations, third order polynomials were plotted through the nodal results from the fibrous PDL model in the alveolar bone in figure 5, 6 and 7.

Figures 5, 6, and 7 compare the nodal strain results from the solid and fibrous PDL models for the three load cases. For all graphs of alveolar bone strain, it can be seen that including the PDL fibres in the model affects the results. Similarly, the PDL fibres affect strain in the trabecular bone region for both of the orthodontic load cases. Conversely, for the occlusal load, strain in the trabecular bone region appears unaffected by the inclusion of the fibres.

In the case of the occlusal load (figure 5), the PDL fibres increase maximum principal strain (the most tensile strain) in the upper region of the alveolar bone when compared to the solid PDL model. This effect is then reversed in the apex region where the PDL fibres reduce the tensile strain. A similar pattern can be seen for minimum principal strain (the most compressive strain) in the alveolar bone where again the PDL fibres increase the strain along the majority of the tooth root apart from the apex region where the strain becomes higher in the solid PDL model. The plots are symmetric because of the symmetry in the loading, but both sides are included for ease of comparison with the plots from the other load cases.

For both orthodontic loads (figures 6 and 7) the strains in the alveolar bone for the solid PDL model are all highest towards the tip of the tooth and then decrease towards the apex. Conversely, for the mesiodistal load (figure 6) the fibrous PDL model shows almost uniform strain on the mesial side of the alveolar bone. A similar pattern is seen with the fibrous PDL model on the buccal side due to the buccolingual load (figure 7) although the strains are not as uniform in this case. Whilst the strains at the apex are always low in the solid PDL model with the orthodontic loads, the strains are usually comparatively high at this location in the fibrous PDL model.

For both orthodontic loads the difference in strain between the solid and fibrous PDL models is most noticeable on the opposite side of the bone to the direction of tooth movement (i.e. on the mesial side in figure 6 and the buccal side in figure 7). In the direction of tooth movement there is little difference between the two models except in the apex region where strain is noticeably higher in the fibrous PDL model.

The buccolingual load is directed through the centre of the tooth and so causes translation of the tooth with very little rotation about its long axis. For this load, the fibrous PDL model allows a greater amount of tipping of the tooth. There is around 30% greater displacement of the tooth crown in this model compared to the solid PDL model along with around 11% decrease in movement of the tooth apex. Conversely, the mesiodistal load causes both rotation of the tooth about its long axis as well as mesiodistal translation. Compared to the solid PDL model, the fibrous PDL model allows around 31% increase in axial rotation at the tooth crown as well as 56% greater displacement of the tooth apex.

## Discussion

Comparing the predicted maximum and minimum principal strains between the solid and fibrous PDL model reveals that inclusion of the PDL fibres influences the strain in the alveolar bone for both occlusal and orthodontic loads. The influence of PDL fibres is not so pronounced in the trabecular bone region, particularly in the occlusal load case where there is very little difference between the results for the two models.

The oblique orientation of the principal fibres in the PDL means they have a function as a suspensory ligament supporting the tooth root within the alveolar bone. Consequently, vertical forces on the tooth may be transmitted as lateral tension to the alveolar wall [22]. This also helps to prevent high stresses occurring at the apex of the tooth root. However, if the PDL is modelled as a continuous solid structure these effects may be lost. Figure 5 shows that the effect of the PDL fibres on the distribution of strain from the tooth to the alveolar bone is noticeable between these two models. At both the inside and outside surfaces of the alveolar bone, the tensile strain is higher for the fibrous PDL model in the upper section of the bone and then lower towards the apex, as would be expected. Although the PDL fibres have been included in this model in a highly schematic representation of their real structure, they do appear to be functioning as predicted. We, therefore, agree with Qian *et al*. [23] who concluded that it may be better to include simplistic PDL fibres than not to include the fibres at all.

However, although the fibres had a substantial influence on the strains observed in the alveolar bone, almost no difference was observed between the two models in the trabecular bone region. The magnitudes of the strains in this region were also far higher than would be expected. Frost [10] suggested that bone modelling occurs at approximately 1500–3000 *µε*. However, the strains recorded in the trabecular bone in our models were around a full order of magnitude greater than that. It is likely that both of these problems are due to the highly simplified geometry and material properties in this model. Since this is a simplified model, the actual strain values are not the primary concern but rather the relative differences between the two models is of interest. Altering Young's modulus of the trabecular bone may decrease the trabecular bone strain but it would have little effect on the behaviour of the tooth in its socket. Similarly, it is possible that the effects of the PDL fibres would be different in the trabecular bone region if the geometry of the trabecular network had been considered.

For both orthodontic loads, the strains at the apex of the tooth socket, on both the inside and outside surfaces, were higher for the fibrous PDL model than for the solid PDL model (Figures 6 and 7). This is the opposite of what was observed in this region for the occlusal load where the fibrous PDL model showed less strain. This is understandable since the fibres only exert force in tension, not in compression. When a vertical load is applied the fibres at the apex are not stretched and so only the soft PDL matrix resists the tooth at this point. However, when an orthodontic load is applied, translation and rotation of the tooth occur which would stretch the apical fibres causing them to exert a force on the alveolar bone at this location.

For both orthodontic load cases, strain in the solid PDL model is always highest at the top of the alveolar bone and then decreases towards the apex of the tooth on both sides of the model. This is not the case for the fibrous PDL model. In this model, there is a more uniform distribution of strain along the length of the alveolar bone on the side of the tooth away from the direction of tooth movement. This is particularly obvious with the mesiodistal load. On the side to which the tooth is moved, there is less difference between the solid and fibrous PDL models except at the apex. This is due to the fact that the PDL fibres only exert force in tension and so on this side the fibrous PDL behaves similarly to a solid PDL.

Compared to the solid PDL model, the fibrous PDL model allows for a greater amount of tooth movement with both orthodontic loads. The crossed structure of the PDL fibres (figure 2) ensures that some fibres are always in tension and therefore resisting tooth movement, regardless of the direction of an applied load [21]. However, this also means that some fibres are in compression and thus not playing any role in resisting the tooth movement. It is therefore not surprising that the fibrous PDL model allows for greater rotation and tipping of the tooth compared to the solid PDL model.

The strain results in figures 6 and 7 can be interpreted in light of the three hypotheses shown in figure 1. Neither set of results agree with the pressure-tension hypothesis. This hypothesis predicts that there should be compression on the side to which the tooth is moved and tension on the opposite side. Neither set of results show distinct regions of compression and tension as predicted.

The alveolar bending hypothesis predicts both compression and tension occurring in the bone on each side of the tooth but on opposite surfaces of the alveolar bone, i.e. tension on the inside surface and compression on the outside surface or vice versa. However, for both the mesiodistal and buccolingual orthodontic loads (figures 6 and 7) the type of stress occurring, either compressive or tensile, is the same at the inside and outside surfaces of the alveolar bone on both sides of the tooth. This suggests that perhaps the bone is not bending as predicted. Alternatively, since the strain results are very similar at the inside and outside surfaces, this may be due to the alveolar bone being very thin in this model. Consequently, the bending may involve the trabecular bone and so the sign change, e.g. from compression to tension, may occur in this region rather than in the alveolar bone region. This appears to be the case for the buccolingual load (figure 7) where on the lingual side the alveolar bone is predominantly in tension while the trabecular bone is slightly more in compression, and on the buccal side the alveolar bone is in compression while the trabecular bone is in tension.

The mesiodistal load causes both tilting of the tooth in the mesiodistal direction and rotation of the tooth about its long axis which is not taken into consideration in the alveolar bending hypothesis (figure 1). A closer inspection of the direction of strains in the alveolar bone indicates that the tensile strains on the distal side and the compressive strains on the mesial side (which are predicted by the alveolar bending hypothesis) are predominantly orientated in the axial direction (i.e. parallel to the long axis of the tooth). This suggests that if the model was only being considered in two dimensions, the alveolar bending hypothesis may appear correct. However, in three dimensions, simple cantilever bending of the alveolar bone is not the only cause of strain in the alveolar bone.

For the buccolingual load, the lingual side is predominantly in tension as predicted. Additionally, the tensile strains are mostly orientated in the axial direction. Conversely, the compressive strains, which are not predicted by the alveolar bending hypothesis, are orientated in the hoop (i.e. directed around the tooth within the horizontal plane) and radial (i.e. directed away from the long axis of the tooth within the horizontal plane) directions. Similarly, on the compression side, the compressive strains are mostly orientated in the axial direction whereas the tensile strains on that side are mostly in the hoop and radial directions. These results also provide some support for the alveolar bending hypothesis, although again strain must be considered in all directions.

Unlike the pressure-tension hypothesis and the alveolar bending hypothesis, the stretched fibre hypothesis does not consider whether the bone is in compression or tension, but is mainly concerned with whether bone is in high strain or low strain. This hypothesis is also the one that should be most affected by whether or not fibres are included in the FE model. For both the mesiodistal load and the buccolingual load, strain is higher in the fibrous PDL model than the solid PDL model on the side which the tooth is moved away from (the mesial side in figure 6 and the buccal side in figure 7). This provides some support to this hypothesis which predicts that strains should be high on this side due stretching of the PDL fibres.

Whilst there is some support for the stretched fibre hypothesis on the side which bone is formed, it is not clear whether resorption on the other side is due to low strains. For the buccolingual load the magnitude of strain on the lingual side is not greatly different to that on the buccal side. For the mesiodistal load there is almost uniform strain on the mesial side (the side from which the tooth is moved away). Conversely, on the distal side (the side to which the tooth is moved) the strains are reasonably low in the upper portion of the alveolar bone but get much higher towards the apex. In neither case are the strains particularly low in the direction of tooth movement to suggest that bone resorption is due to under-loading.

For both orthodontic loads, the strains observed in the alveolar bone are all far below those values typically thought to cause bone remodelling [10]. However, it should be emphasised that this is a simplified model and so the absolute values of strain in the model cannot be assumed to be accurate. Nevertheless, this agrees with the results of other authors who also found very low strains in the alveolar bone (e.g. [3], [15]). The low strains commonly observed in the alveolar bone have led to the proposal that the stimulus for orthodontic tooth movement comes from the PDL rather than the alveolar bone (e.g. Chen *et al*. [12]). However, if the PDL is responsible for controlling orthodontic tooth movement, the mechanical stimulus for this is still unclear. For example, Qian *et al*. [47] used normal strain in the PDL to drive tooth movement whereas Chen *et al*. [12] chose hydrostatic stress in the PDL.

It has been suggested that, rather than all bones responding to the same levels of strain, it may be the case that resorption and formation are triggered at different strain levels in different bones [48]. Also, the alveolar bone is thought to be one of the most physiologically active bones in mammals [49]. Therefore, whilst the strain in the alveolar bone may not be at the level known to cause remodelling in other bones, it may still be sufficiently high to cause remodelling here. Therefore, the fact that strains are low in the alveolar bone does not eliminate the possibility that the alveolar bone itself provides the stimulus for orthodontic tooth movement.

The results from these models are inconclusive in regards to which of the proposed hypotheses for orthodontic tooth movement may be correct. The results do not appear to provide any support for the pressure-tension hypothesis but provide some support for each of the other two. Regions of compression and tension correspond roughly to where they would be expected from the alveolar bending hypothesis. The results also show that including the PDL fibres is particularly important for increasing strain on the bone formation side as predicted by the stretched fibre hypothesis. It is possible that the exact mechanism responsible for orthodontic tooth movement is a combination of these two hypotheses along with regulation from the PDL itself.

These results show that including the fibrous structure of the PDL are important in FE models when investigating orthodontic loads. Compared to the fibrous PDL model, the solid PDL model restricts the amount of tooth movement observed. The fibres also help to distribute the load throughout the alveolar bone compared to the solid PDL model where most of the load is supported by the upper portion of the bone. Unsurprisingly, the fibres have most effect on the side of the bone from which the tooth is moved away since they will mostly be in tension.

Of course, care must be taken when interpreting these results due to the highly simplified geometry and material properties in the model. However, as we were mainly concerned with the relative difference between the results from two models, the results clearly show that including the PDL fibres influences both the magnitude and distribution of the strain produced in the surrounding bone. With the limitations of this model in mind, it will be interesting to investigate the role of PDL fibres in morphologically accurate models based on microCT scans of real human mandibles.

## Supporting Information

File S1 Model testing.
(PDF)
