# Supplementary material for: The Biomechanical Function of Periodontal Ligament Fibres in Orthodontic Tooth Movement
Source: PLoS One. 2014 Jul 18;9(7):e102387. doi: 10.1371/journal.pone.0102387 (PMC4103804; doi:10.1371/journal.pone.0102387)
Supplement: File S1 — Model testing. (PDF) [file pone.0102387.s001.pdf]

Supplementary material for the article "The biomechanical function of periodontal ligament fibres in orthodontic tooth movement"

Steven W. McCormack[1], Ulrich Witzel[2], Peter J. Watson[1], Michael J. Fagan[1], Flora Gröning[3]*

[1] Medical and Biological Engineering Research Group, School of Engineering, University of Hull, Hull HU6 7RX, UK
[2] Fakultät für Maschinenbau, Ruhr-Universität Bochum, Universitätsstraße 150, 44801 Bochum, Germany
[3] Musculoskeletal Research Programme, School of Medicine and Dentistry, University of Aberdeen, Aberdeen AB25 2ZD, UK
* Corresponding Author, E-mail: f.groening@abdn.ac.uk

Model Testing

A number of tests were carried out to assess the suitability of this idealised single tooth model. First, a model without a PDL was developed to highlight the importance of including the PDL in dental FE models. As well as testing the results from a no PDL model, four key issues were investigated with respect to model testing: boundary conditions, mesh convergence, model validation and sensitivity to changes in the PDL. The details of this model testing are given here.

1. No PDL Model

The primary aim of this research is to investigate the significance of including the fibres of the PDL in FE models. However, some models do not include PDL at all (e.g. [1, 2]), yet alone model its fibrous structure. Although almost all single tooth models do include the PDL, in order to show the importance of modelling the PDL in dental FE models, a model was developed without a PDL. This no PDL model was developed from the solid PDL model. The same shape was used but the material assigned to some of the volume meshes was changed. The tooth, cortical shell and trabecular bone region from the solid PDL model were left the same. In order to remove the PDL, the PDL volume from the solid PDL model was remeshed to be alveolar bone, while the alveolar bone volume was remeshed to be trabecular bone. The boundary conditions were left the same and a 500 N occlusal load was applied. The results from the no PDL model were then compared to the solid PDL model to show the importance of including a PDL. To compare the results, the trabecular bone region was cut in the buccolingual direction and then the strains in this region were compared visually for the two models.

Figure S1 shows the strain in the mandibular bone for the no PDL and solid PDL models. From visually comparing the two plots it can be seen that including the PDL in models alters both the magnitude and distribution of strain in the trabecular bone region especially in the region surrounding the alveolar bone. The presence of the PDL reduces the strain around the whole of the alveolar bone, especially in the alveolar crest and apex regions. Therefore, the results from the no PDL model confirmed that the presence of the PDL is important in order to reduce the local bone strain in the region surrounding the tooth root.

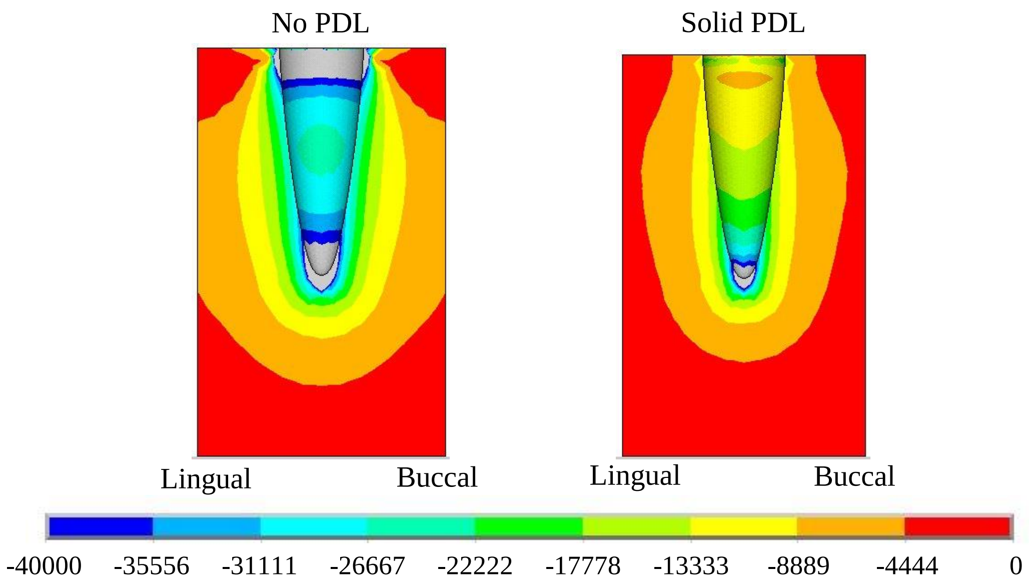

Figure S1. Contour plots showing the minimum principal microstrain in the trabecular bone region of the no PDL and solid PDL models.

2. Boundary Conditions Testing

Boundary conditions applied to FE models have the potential to greatly influence the results obtained. The boundary conditions applied around the edges of the single tooth model were designed to replace the rest of the mandibular bone which has not been modelled. In order to test whether or not the boundary conditions applied are appropriate, two additional “U-shaped” models were developed: a solid PDL U-shaped model and a fibrous PDL U-shaped model. The fibrous PDL U-shaped model is shown in figure S2. As in the case of the single tooth model, the only difference between these two U-shaped models is how the PDL is modelled.

To create the U-shaped models, additional bone was added to the original models to approximately represent the remainder of the mandible. The dimensions of the U-shaped models were chosen to approximate the size and shape of a typical human mandible from a superior view (figure S3). For the U-shaped models, the single tooth model was assumed to represent the first lower right premolar. The additional bone gave the model a further 20,904 elements. These elements included 17,522 higher order tetrahedral elements (SOLID187) and 3,382 shell elements (SHELL281). The total number of elements in the solid PDL U-shaped model was 123,758, with there being an additional 448 link elements (LINK10) in the fibrous PDL model. A 500 N occlusal load was applied in the same way as the single tooth model. All nodes on the two end faces of the model, which are away from the region of interest around the tooth, were fixed in all degrees of freedom. The loading and boundary conditions applied to the model can be seen in figure S2.

In order to test the influence of the boundary conditions on the single tooth model, results from the single tooth model were compared to those from the U-shaped model for both solid and fibrous PDL. Two sets of results were compared: firstly, the vertical tooth displacement (shown in table S1); and secondly, the minimum principal strain in the trabecular bone region of the single tooth model and the corresponding region in the U-shaped model (shown in figures S4 and S5 for solid and fibrous PDL respectively).

The results in table S1 show that the vertical tooth displacement, defined as the change in distance between the most apical node on the tooth root and the corresponding node on the alveolar bone, was very similar between the two models: for both solid and fibrous PDL there was less than 2% reduction in the vertical tooth displacement for the U-shaped model compared to the original model. Due to bending of the additional bone, the overall nodal displacements are larger for the U-shaped model. However, it is the local tooth displacement which is of concern here and the results show this is not greatly affected by the boundary conditions.

The minimum principal strain results (figures S4 and S5) show that the boundary conditions do influence the results in the regions near to them. In both figures, S4 and S5, the top row of plots illustrate that the boundary conditions influence both the magnitude and distribution of strain in the outer regions of the trabecular bone. However, the bottom row in both figures show that in the region of interest, near to the alveolar bone, there is little difference between the two models, suggesting that the boundary conditions do not adversely affect the results here.

From these tests it can be concluded that the boundary conditions applied to the simplified section of mandible are acceptable for the purposes of this study. As expected, the boundary conditions applied alter results in the region of the tooth model near to them. However, they do not greatly influence the local tooth displacement or the strain in the region of interest near to the alveolar bone.

Table S1. Vertical tooth displacement results for original models and u-shaped mandible models.

**Test**	**Model**	**Vertical Displacement (mm)**			**Width of Tooth Socket after Displacement (mm)**	**Vertical Tooth Displacement (mm)[a]**
		**Tooth Apex**	**PDL Apex**	**Tooth Socket Apex**		
Solid PDL	Original	-0.205	-0.134	-0.134	0.200	0.070
	U-Shaped	-0.448	-0.379	-0.378	0.200	0.069
Fibrous PDL	Original	-0.207	-0.137	-0.137	0.200	0.070
	U-Shaped	-0.451	-0.381	-0.381	0.200	0.069

[a] Katona & Qian [3]

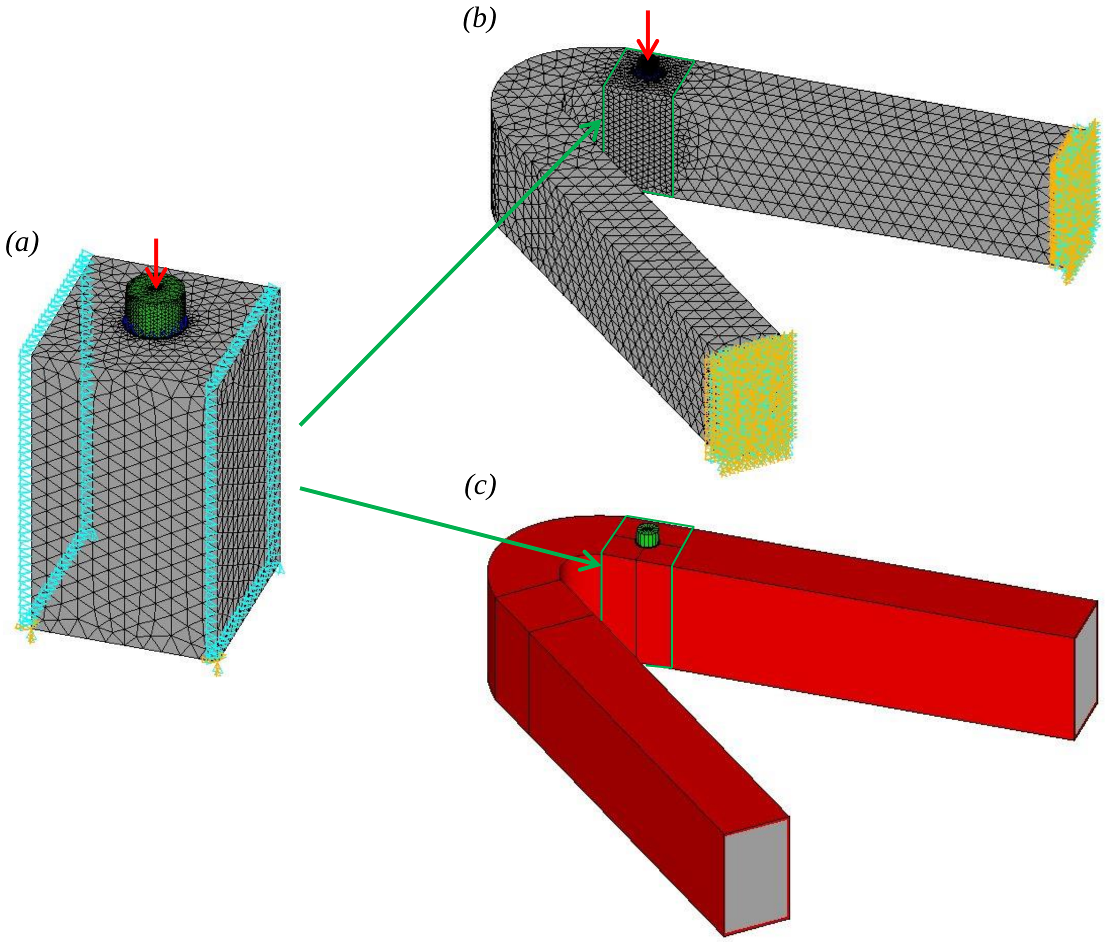

Figure S2. Original single tooth model and U-shaped tooth model: (a) original single tooth model showing load and boundary conditions; (b) finite element mesh for U-shaped model showing the location of the original model within the U-shaped model along with the vertical occlusal load and boundary conditions applied; (c) area plot showing areas meshed with shell elements.

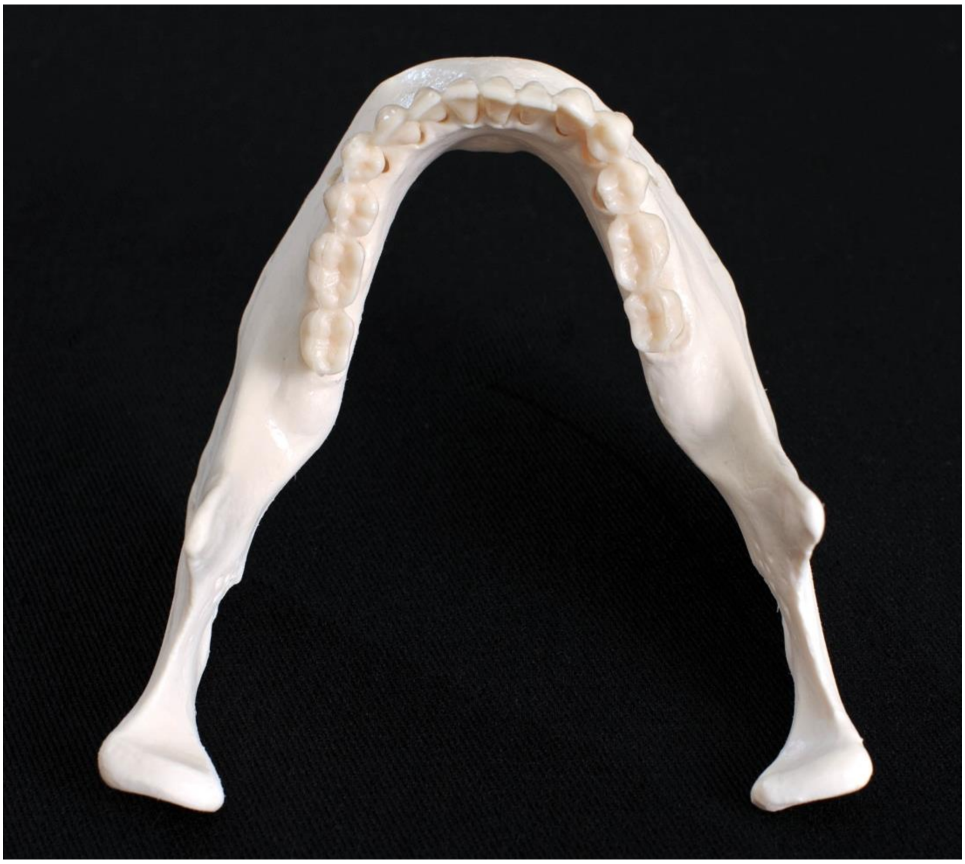

Figure S3. Superior view of a replica human mandible used to approximate the dimensions for the U-shaped models.

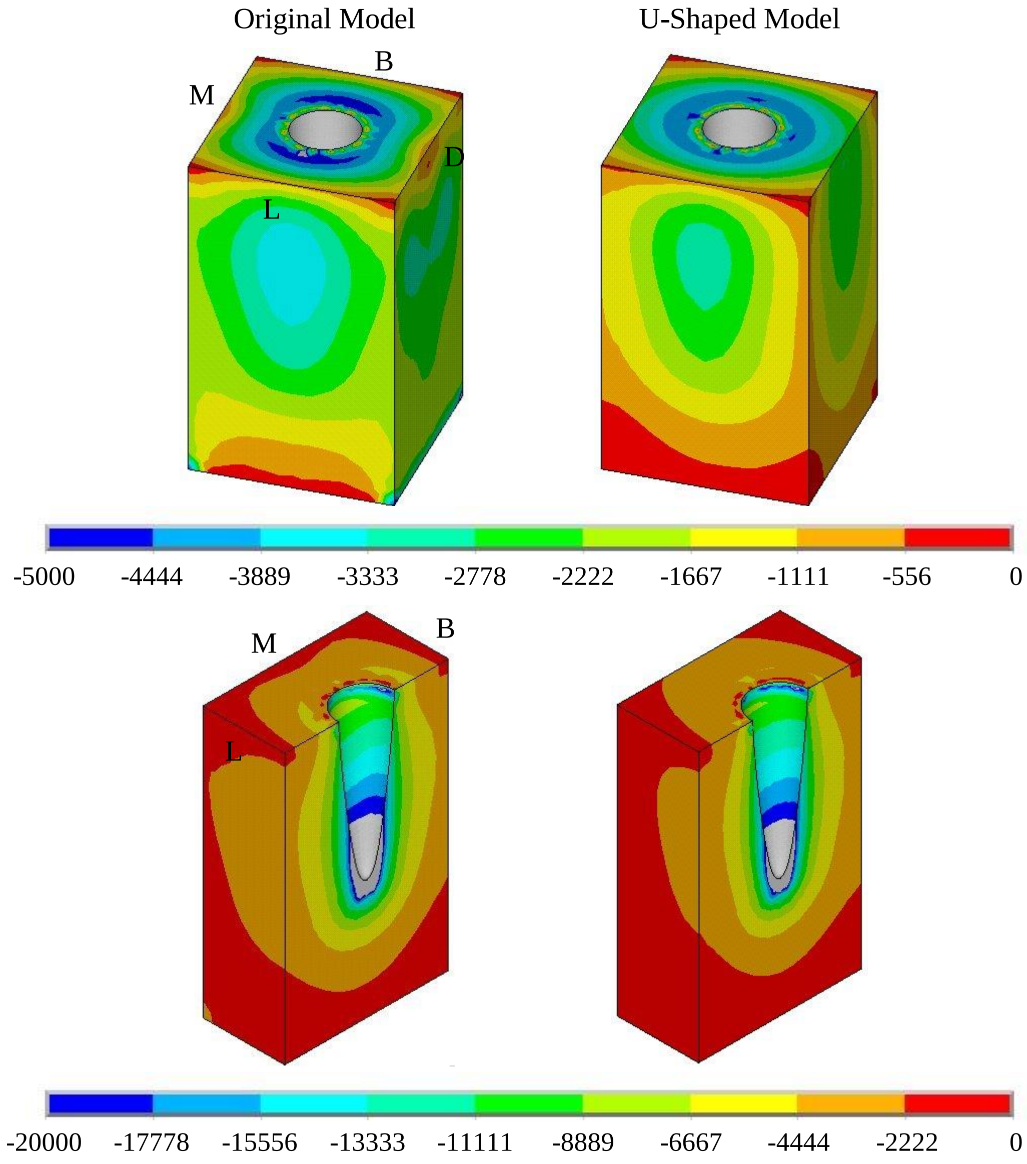

Figure S4. Solid PDL model: the minimum principal microstrain distribution in the trabecular bone region of the original single tooth model and the corresponding region of the U-shaped model for the solid PDL models. Directions are shown only on the original model plots but can be inferred for the U-shaped model plots. Directions are represented by M for mesial, D for distal, B for buccal and L for lingual.

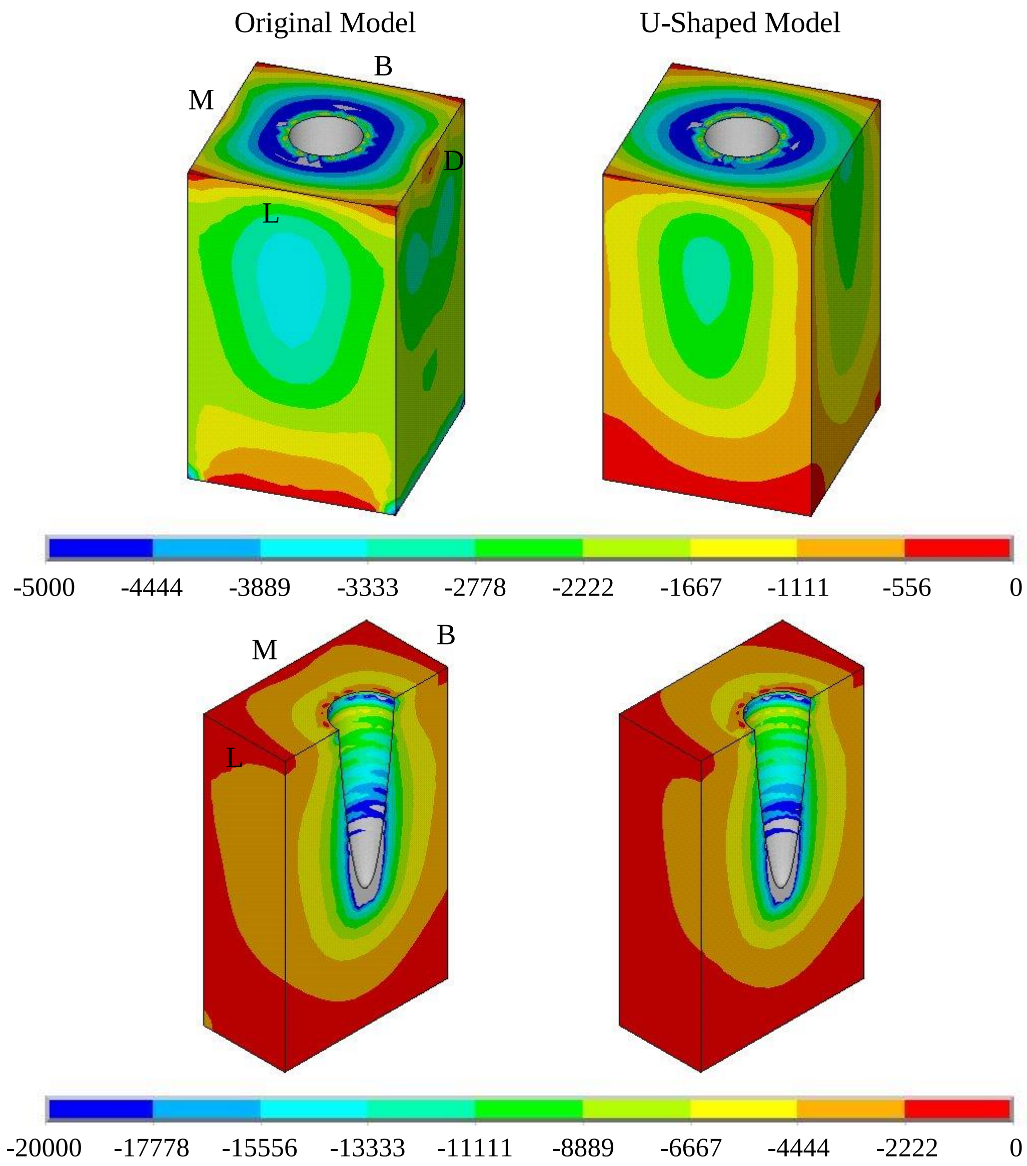

Figure S5. Fibrous PDL model: the minimum principal microstrain distribution in the trabecular bone region of the original single tooth model and the corresponding region of the U-shaped model for the fibrous PDL models. Directions are shown only on the original model plots but can be inferred for the U-shaped model plots. Directions are represented by M for mesial, D for distal, B for buccal and L for lingual.

3. Mesh Convergence Test

A mesh convergence test was also performed for both solid and fibrous PDL models. The model was remeshed using a smaller element size thus increasing the number of elements in the model from approximately 100,000 to just over 1.9 million. The resulting tooth displacements only varied by around 1% compared to the original models and thus it could be concluded that the models had converged.

4. Model Validation

Since this is a very simplified model, stresses and strains would be expected to differ from those in a real tooth and so direct validation of these against experimental results is not possible. However, it would still be expected that the tooth displacement observed would be comparable to previously published data. Therefore, to validate the model, tooth displacement results (0.0703mm) were compared to those reported in literature and they were seen to be of a similar magnitude (e.g. 0.12mm by Borák *et al.* [4] and 0.3mm by Ichim *et al.* [5]).

5. Sensitivity Analysis

A sensitivity analysis was carried out to assess the effect of altering the material properties assigned to the PDL component using the fibrous PDL model. Five values required to specify the PDL were tested, namely: Young's modulus and Poisson's ratio of the solid component, and Young's modulus, cross-sectional area and initial strain of the link elements. The results of this are shown in table S2.

The sensitivity analysis results show that the value assigned to Poisson's ratio of the solid PDL component does not have much influence on the results. Altering the value assigned to Young's modulus of the solid PDL component has more influence on the results as the value gets higher. A 10 times increase in this value from 0.1 MPa to 1 MPa causes approximately a 9% reduction in the vertical tooth displacement, whereas a further 10 fold increase to 100 MPa causes almost a 90% reduction in displacement. Likewise, the influence of adjusting the Young's modulus of the link elements becomes greater as its value increases. Both the cross sectional area and the initial strain assigned to the link elements also have an important influence on the tooth displacement. Doubling the cross sectional area approximately halves the tooth displacement and a 10 times increase in initial strain causes almost a 60% reduction in tooth displacement.

Adjusting the values of Young's modulus for the solid PDL component, Young's modulus of the link elements, the cross sectional area of the link elements or the initial strain of the link elements, all had a noticeable influence on the vertical tooth displacement. The influence of Young's modulus of both the solid component and link elements increased as their value increased. The solid component in this model represents the soft matrix surrounding the collagen fibres in the physiological PDL and so its

Young's modulus is likely to be well below that of the fibres. Therefore, the value chosen for the matrix may be less important than the values assigned to the fibres when fibres are modelled. It would appear particularly important to choose appropriate values for the cross sectional area, initial strain and Young's modulus of the link elements. However, since there are very little reliable data available from which to choose these, it is important to conduct sensitivity analyses when including PDL fibres in future FE models.

Table S2. Results from sensitivity analysis with fibrous PDL model (numbers in bold represent the original model with the material properties shown in table 1).

**Test**	**Value**	**Vertical displacement (mm)**			**Width of tooth socket after displacement (mm)**	**Vertical tooth displacement (mm)[a]**
		**Tooth apex**	**PDL apex**	**Tooth socket apex**		
Young's modulus of PDL Matrix (E_{PDL})	E_{PDL} = 0.1	-0.215	-0.138	-0.138	0.200	0.077
	**E_{PDL} = 1**	**-0.207**	**-0.137**	**-0.137**	**0.200**	**0.070**
	E_{PDL} = 10	-0.169	-0.133	-0.133	0.200	0.036
	E_{PDL} = 100	-0.128	-0.123	-0.123	0.200	0.005
Young's modulus of PDL fibres (E_{LINK})	E_{LINK} = 30	-0.861	-0.139	-0.139	0.200	0.722
	E_{LINK} = 300	-0.346	-0.140	-0.140	0.200	0.206
	**E_{LINK} = 1 000**	**-0.207**	**-0.137**	**-0.137**	**0.200**	**0.070**
	E_{LINK} = 3 000	-0.157	-0.132	-0.132	0.200	0.025
Poisson's ratio of PDL matrix (ν_{PDL})	ν_{PDL} = 0.3	-0.208	-0.137	-0.137	0.200	0.071
	**ν_{PDL} = 0.45**	**-0.207**	**-0.137**	**-0.137**	**0.200**	**0.070**
	ν_{PDL} = 0.49	-0.203	-0.137	-0.137	0.200	0.066
Cross sectional area of link elements (CSA_{LINK})	CSA_{LINK} = 0.04	-0.241	-0.138	-0.138	0.200	0.103
	**CSA_{LINK} = 0.06**	**-0.207**	**-0.137**	**-0.137**	**0.200**	**0.070**
	CSA_{LINK} = 0.08	-0.189	-0.136	-0.136	0.200	0.053
Initial strain of link elements (ε_0)	**ε_0 = 0**	**-0.207**	**-0.137**	**-0.137**	**0.200**	**0.070**
	ε_0 = 0.01	-0.190	-0.136	-0.136	0.200	0.054
	ε_0 = 0.1	-0.114	-0.092	-0.093	0.201	0.022

[a] Katona & Qian [3]

References

1. Marinescu R, Daegling DJ, Rapoff AJ (2005) Finite-Element Modeling of the Anthropoid Mandible: The Effects of Altered Boundary Conditions. Anat Rec 283A: 300-309.
2. Boryor A, Geiger M, Hohmann A, Wunderlich A, Sander C, et al. (2008) Stress distribution and displacement analysis during an intermaxillary disjunction – A three-dimensional FEM study of a human skull. Journal of Biomechanics 41: 376-382.
3. Katona TR, Qian H (2001) A mechanism of noncontinuous supraosseous tooth eruption. Am J Orthod Dentofacial Orthop 120: 263-271.
4. Borák L, Florian Z, Bartáková S, Prachár P, Murakami N, et al. (2011) Bilinear elastic property of the periodontal ligament for simulation using a finite element mandible model. Dental Materials Journal 30(4): 448-454.
5. Ichim I, Kieser JA, Swain MV (2007) Functional significance of strain distribution in the human mandible under masticatory load: Numerical predictions. Archives of Oral Biology 52: 465-473.
